# Editorial: From Loneliness to Belonging - The Path We Hope to Take

**DOI:** 10.3389/fpsyg.2021.761440

**Published:** 2021-10-05

**Authors:** Ami Rokach, Joel O. Goldberg

**Affiliations:** ^1^Department of Psychology, The Center for Academic Studies, Or Yehuda, Israel; ^2^Department of Psychology, York University, Toronto, ON, Canada

**Keywords:** belonging, human connection, loneliness, social isolation, pandemic

The theme of our special issue, from loneliness to belonging, has resonated during the global pandemic, as so many have been left isolated due to tragic loss and due to mandated social restrictions. The path that we hope to take in order to emerge from loneliness is toward belonging; having someone who cares deeply about us is essential (Rokach, 2017). With many glued to television-watching during COVID-19 isolation, one phenomenon has been the popularity of the award-winning comedy show, Ted Lasso, a coach who brings inspiration and optimism to his team amidst their sense of isolation and despair, with many of us resonating to his words “I promise you, there is something worse out there than being sad. And that is being alone and being sad. Ain't no one in this room alone” (Longshore, 2020).

Indeed, in a seminal article highlighting the importance of belonging, Baumeister et al. (2002) suggested that we have an inescapable drive to form and keep lasting and positive relationships. But there is more. A critical point, when discussing belonging is that social relationships are important, but it is their quality rather than quantity which makes the difference (see Rokach, 2014), that we can feel alone even in a room of people, that we also have to feel we matter. A number of papers in our special issue explore, from both empirical and theoretical perspectives, the importance of empathy and the importance of mattering in emerging from loneliness to journey on this path toward belonging.

It is not just during a pandemic health crisis that these issues are relevant. Our Western culture has aspects which appear to magnify the alienation and impose the separateness that we can feel (Rokach, 2004), although as individuals we still long to belong and to be loved. Segrin et al. (2016) emphasized that from the moment of birth, our welfare depends on the care of others; evolutionarily speaking, our ability to survive begins with and over time remains dependent upon successful affiliation with other people. And on the other hand, when people are socially isolated or marginalized (Rokach, 2014), we experience despairing loneliness which motivates us to heal through re-establishing social contacts and belonging to a community. Loneliness has been found to have a significant impact on the person's physical health and emotional well-being (Ben-Zur, 2013). Loneliness may potentially negatively affect one's physical, emotional and psychosocial functioning (Cacioppo et al., 2015). Amidst loneliness may emerge alcoholism (Åkerlind and Hörnquist, 1992), depression (Cacioppo et al., 2003), social anxiety (Kearns et al., 2015), cognitive decline and even suicidal ideation (Wilson et al., 2007). Moreover, loneliness may exacerbate obesity, elevate blood pressure, and hasten mortality (Holt-Lunstad et al., 2015). Loneliness is, then, a powerful mental and physical health consideration, and obviously needs to be addressed on a personal, as well as on a community level. Research has indeed confirmed the Ted Lasso admonition that there is something worse than being sad, that is being alone and sad. And in turn, research has also celebrated the renewing power of social integration by suggesting that belonging can counteract, in its' positive influence, the deleterious impacts of cigarette smoking, obesity, elevated blood pressure, and physical inactivity (Cacioppo et al., 2015).

This special issue was composed of seven articles, tracing various aspects of the pathway from loneliness to belonging.

Hu et al. focused on the relationship of loneliness to differential aspects of empathy. Results indicated that compared to non-lonely people, lonely people were more likely to choose positive empathy but to avoid negative empathy.

Similarly addressing loneliness and empathy, Berduzco-Torres et al., found that family loneliness showed an inverse correlation with empathy, teamwork, and learning measures, thus confirming how family loneliness is detrimental to the development of medical professionalism.

McComb et al. set out to explore how loneliness relates to a construct termed mattering, which is the sense of feeling important to other people. They concluded, based on their findings, that feeling as though one does not matter to others is associated with increased state and trait loneliness among young women.

Liu et al. focused on loneliness of those suffering from severe mental illnesses [SMIs]. The objective of this cross-sectional study was to examine the influence of health literacy and social support on the loneliness of patients with SMI. Findings suggest psychoeducation for SMI patients, and for their informal caregivers, may offer beneficial effects toward reducing loneliness in this vulnerable population.

Victor conducted a review to uncover the extent to which longitudinal studies of loneliness may point to negative health outcomes in dementia. Her findings suggested that the evidence to support a relationship between loneliness and dementia is inconclusive largely because of methodological limitations of existing studies.

Rokach et al. explored the qualitative aspects of the loneliness experienced by the blind and visually challenged. Remarkably, their findings pointed out that when comparing the blind to the general population, the two populations differed significantly in their scores on four of the five loneliness subscales (except emotional alienation), but in the opposite direction of what was expected. The authors opined that the visually impaired scored lower on loneliness measures due to transcending their blindness, and connecting with those around them in different—and not necessarily less meaningful-manner than the seeing general population.

Miller focused, in his opinion piece, on the various implications that the COVID-19 pandemic may have especially on loneliness, amidst social, economic, and political crises. Miller observed that an alarming rise in domestic violence was noted due to COVID-19, and more generally, the pandemic may serve to adjust our appraisals of others as t a reminder of the fragility of our lives and the importance of maintaining health.

Taken together, the special issue provides a theory and research space to remind us of the journey to emerge from loneliness to belonging, to remind us that no one in this room is alone.

## Author Contributions

All authors listed have made a substantial, direct and intellectual contribution to the work, and approved it for publication.

## Conflict of Interest

The authors declare that the research was conducted in the absence of any commercial or financial relationships that could be construed as a potential conflict of interest.

## Publisher's Note

All claims expressed in this article are solely those of the authors and do not necessarily represent those of their affiliated organizations, or those of the publisher, the editors and the reviewers. Any product that may be evaluated in this article, or claim that may be made by its manufacturer, is not guaranteed or endorsed by the publisher.
